# Prediction of Critical Care Outcome for Adult Patients Presenting to Emergency Department Using Initial Triage Information: An XGBoost Algorithm Analysis

**DOI:** 10.2196/30770

**Published:** 2021-09-20

**Authors:** Hyoungju Yun, Jinwook Choi, Jeong Ho Park

**Affiliations:** 1 Interdisciplinary Program of Medical Informatics College of Medicine Seoul National University Seoul Republic of Korea; 2 Department of Biomedical Engineering College of Medicine Seoul National University Seoul Republic of Korea; 3 Institute of Medical and Biological Engineering Medical Research Center Seoul National University Seoul Republic of Korea; 4 Department of Emergency Medicine College of Medicine Seoul National University Seoul Republic of Korea; 5 Laboratory of Emergency Medical Services Biomedical Research Institute Seoul National University Hospital Seoul Republic of Korea

**Keywords:** triage, critical care, prediction, XGBoost, explainable machine learning, interpretable artificial intelligence, machine learning, algorithm, prediction, outcome, emergency, triage, classify, prioritize, risk, model

## Abstract

**Background:**

The emergency department (ED) triage system to classify and prioritize patients from high risk to less urgent continues to be a challenge.

**Objective:**

This study, comprising 80,433 patients, aims to develop a machine learning algorithm prediction model of critical care outcomes for adult patients using information collected during ED triage and compare the performance with that of the baseline model using the Korean Triage and Acuity Scale (KTAS).

**Methods:**

To predict the need for critical care, we used 13 predictors from triage information: age, gender, mode of ED arrival, the time interval between onset and ED arrival, reason of ED visit, chief complaints, systolic blood pressure, diastolic blood pressure, pulse rate, respiratory rate, body temperature, oxygen saturation, and level of consciousness. The baseline model with KTAS was developed using logistic regression, and the machine learning model with 13 variables was generated using extreme gradient boosting (XGB) and deep neural network (DNN) algorithms. The discrimination was measured by the area under the receiver operating characteristic (AUROC) curve. The ability of calibration with Hosmer–Lemeshow test and reclassification with net reclassification index were evaluated. The calibration plot and partial dependence plot were used in the analysis.

**Results:**

The AUROC of the model with the full set of variables (0.833-0.861) was better than that of the baseline model (0.796). The XGB model of AUROC 0.861 (95% CI 0.848-0.874) showed a higher discriminative performance than the DNN model of 0.833 (95% CI 0.819-0.848). The XGB and DNN models proved better reclassification than the baseline model with a positive net reclassification index. The XGB models were well-calibrated (Hosmer-Lemeshow test; *P*>.05); however, the DNN showed poor calibration power (Hosmer-Lemeshow test; *P*<.001). We further interpreted the nonlinear association between variables and critical care prediction.

**Conclusions:**

Our study demonstrated that the performance of the XGB model using initial information at ED triage for predicting patients in need of critical care outperformed the conventional model with KTAS.

## Introduction

Overcrowding in the emergency department (ED) has become a major worldwide health care problem [1-3]. Therefore, most EDs have a triage to manage growing patient volumes [2,4,5]. ED triage is the first risk assessment for prioritizing patients at high risk and determining the course of ED care for patients [5-8]. It is vital to accurately identify patients who need immediate care at triage and provide rapid care to patients in ED since delay in care may result in increased morbidity and mortality for many clinical conditions [2,4,5,7,9,10].

Five-level triage systems, including the Canadian Triage and Acuity Scale (CTAS), Manchester Triage System (MTS), and emergency severity index (ESI), are widely used [2,8,9]. The Korean Triage and Acuity Scale (KTAS) was developed in 2012 based on CTAS and has been used nationally as the ED triage tool in Korea since 2016 [11-13]. Although five-level triage systems are well established in ED, they need to be improved because they heavily rely on healthcare providers’ subjective judgment, resulting in high variability [5,7-10,12].

Machine learning algorithms such as extreme gradient boosting (XGB) and deep neural networks (DNNs) have the advantage of fitting nonlinear relationships between predictors and outcomes in large data sets [10,14-17]. Recent literature has shown machine learning prediction models using triage information perform better than the baseline model using the conventional approach of the five-level triage score for screening ED patients at risk of hospitalization, intensive care unit (ICU) admission, mortality, and critical care, which is defined as the combined outcome of ICU admission and mortality [3,6-10,12,17-20].

Clinical prediction models should be characterized by discrimination, which indicates how well the model differentiates patients who will have an event from those who will not, and by calibration, which refers to the agreement between predictions and the observed outcome [20-23]. Systematic reviews have reported that machine learning model studies for clinical predictions almost always assessed discriminative performance using the area under the receiver operating characteristic (AUROC) curve, and the reliability of risk prediction, namely calibration, was rarely evaluated [24-27]. In most of the previous studies for triage in ED, performance metrics pertaining to discriminating power were provided, but calibration, which assesses how close the prediction is to the true risk, was rarely reported. Raita et al provided the AUROC of ED triage prediction of critical care outcomes using four machine learning algorithms [9]. Kwon et al evaluated the discrimination of deep learning–based triage and acuity score model for critically ill patients [12]. Goto et al [10] investigated the discriminative performance of machine learning approaches for predicting critical care outcomes for patients with asthma and chronic obstructive pulmonary disease exacerbations in the ED. However, the calibration of the models for critical care outcomes was not included as a performance measure in the studies reviewed. Poorly calibrated prediction algorithm models can be misleading, which may result in incorrect and potentially harmful clinical decisions [24,26-28]. Therefore, a study including a performance evaluation of calibration in the prediction model for patients with a critical illness at triage in ED is required.

Moreover, no study has investigated the interpretability of machine learning models for the triage in ED to date. The interpretability of machine learning is defined as the degree to which the machine learning user understands and interprets the prediction made by a machine learning model [14-16]. The lack of interpretation is the barrier to establishing clinicians’ trust and the broader adoption of machine learning models in clinical practices [14,15,29]. Explaining the justification of prediction outcomes of the machine learning algorithm model ensuring that the model makes the right predictions for the right reasons is required to enhance clinicians’ buy-in [14-16,29]. Therefore, in this study, we apply the partial dependence plot (PDP), a global model-agnostic technique for explaining the relationship between predictors and prediction results, to investigate the interpretability of machine learning prediction for clinical care in ED [15,16].

We developed and validated the machine learning prediction model for critical care outcomes using routinely available triage information. We hypothesized that applying a machine learning algorithm to ED triage information could improve the performance of critical care outcome prediction for patients who visited an ED compared with the baseline KTAS model using logistic regression.

## Methods

### Study Design, Setting, and Data Source

This was a retrospective study of patients that visited the emergency department of an urban tertiary-care academic center with an annual census of about 70,000 from January 1, 2016, and December 31, 2018. We collected the demographics (age and gender), mode of ED arrival, the time interval between onset and ED visit, reason of ED visit, chief complaint, initial vital sign measurements, KTAS score, and disposition results (ED results and admission results). All data were acquired from the Korean National Emergency Department Information System.

### Study Population

We considered adult patients (aged ≥18 years) who visited an ED during the study period. We excluded patients who did not need clinical outcomes prediction at triage, that is, cardiac arrest or death upon ED arrival. Furthermore, we excluded patients transferred to another hospital or those with uncompleted care because it was impossible to ascertain their ED results. Patients with missing or invalid information at triage were not included (Table S1, Multimedia Appendix 1).

### Outcome

The primary outcome in this study was critical care outcome, defined as the composite of direct admission to ICU or in-hospital mortality following previous studies [4,7,9].

### Variables and Preprocessing

For the prediction of critical care, we included a total of 13 variables: age, gender, mode of ED arrival, the time interval between onset and ED arrival, reason of ED visit, chief complaint, systolic blood pressure (SBP), diastolic blood pressure (DBP), pulse rate (PR), respiratory rate (RR), body temperature (BT), oxygen saturation, and level of consciousness namely, alert, verbal, painful, and unresponsive (AVPU). The mode of ED arrival was categorized into two options as either ambulance use or not. The reason for the ED visit had two values, either illness or injury. The chief complaints, which were based on the Unified Medical Language System (UMLS), were selected from the list of 547 codes. The preprocessing details for the variables are described in Multimedia Appendix 1 (Table S1).

### Model Development

The prediction model of critical outcome was developed by using two modern prediction algorithms: XGB and DNN.

XGB algorithm is a cutting-edge machine learning application of gradient boosting mechanisms [3,8,9,30]. The gradient boosting is an ensemble algorithm with which new trees focus on adjusting errors produced by the previous tree models [8,30-32]. We implemented the XGB model on the training set using five-fold cross-validation. The maximum depth of five and a learning rate of 0.1 were selected from grid search for tuning hyperparameter (Table S2, Multimedia Appendix 1). For a DNN algorithm that equips the learning mechanism to fit nonlinear relationships and high order interactions, [5,10,20,33], we used three hidden layers selected from the grid search: (1) a rectified linear unit as the activation function; (2) an adaptive moment estimation as the optimizer; (3) a drop-out rate of 10%, zero value for lambda, and binary cross-entropy as the loss function (Table S2, Multimedia Appendix 1).

Random sampling was applied to split the entire data set into training (80%) and validation sets (20%). The performance of the prediction model was evaluated in the validation data set.

### Statistical Analysis

For the characteristics of the study population according to critical care, a two-tailed *t* test or Mann–Whitney *U* test was conducted for the continuous variables, and the chi-square test or Fisher’s exact test was performed for the categorical variables.

The discriminating power as a primary measure was evaluated by AUROC, which refers to how well the model differentiates those at a higher risk of having an event from those at lower risk [17,21]. We used the DeLong test to compare AUROC between models [9]. Reclassification improvement was evaluated using the net reclassification index (NRI) [9,10,21]. The NRI quantifies how well a new model reclassifies subjects compared with the reference model [9,10,21]. Model calibration was assessed with the Hosmer-Lemeshow test, a goodness-of-fit measure for prediction models of binary outcomes [20,21,23,34]. Furthermore, the calibration was depicted on a reliability diagram to represent the relationship between predicted probability and observed outcomes [17,20,21,23,34]. The perfect calibration should be in the 45-degree line [17,23,34]. The sensitivity, specificity, positive predictive values (PPVs), and negative predictive values (NPVs) were reported on performance metrics. We used a sensitivity cutoff point of 85% for the illustration of performance.

The variable importance of each prediction model was assessed and determined using the approach of permutation variable importance, which computes the importance by measuring the decrease of model prediction performance (AUROC) when each variable is permuted [35-38].

Finally, for the best prediction model, the PDP was visualized for both the direction and effect size of each variable after averaging out the effect of the other predictors in the model [38-40]. More concretely, the partial dependence by calculating the marginal effect of a single variable on the prediction outcome demonstrates whether the association between a variable and the prediction response is linear or nonlinear [15,40,41].

A two-tailed *P* value of <.05 was considered statistically significant, and a 95% CI was provided. All analyses were performed using the R software (version 3.6.1, R Foundation for Statistical Computing).

### Ethics Statement

The Institutional Review Board of Seoul National University Hospital approved this study, and they waived the requirement for consent. All methods were performed in accordance with the relevant guidelines and regulations.

## Results

### Characteristics of Study Subjects

**Figure 1:**
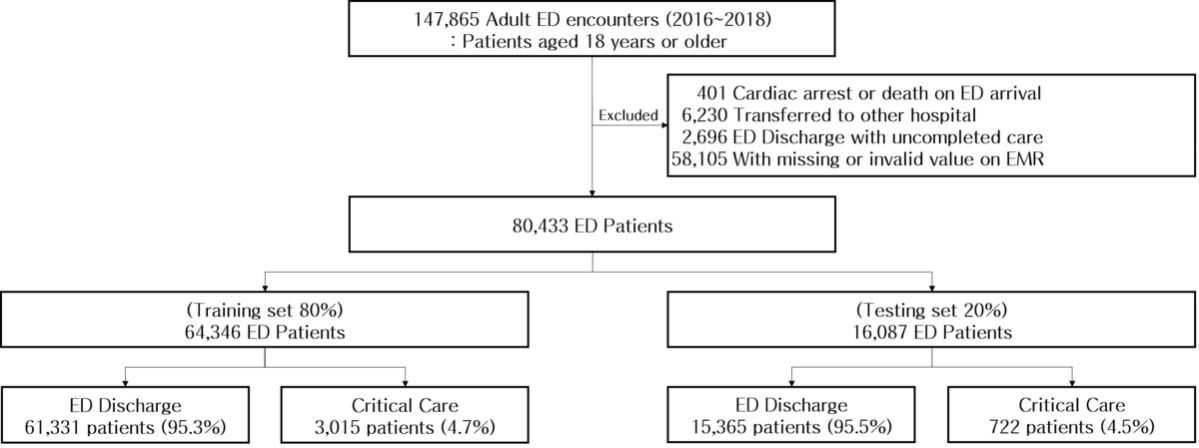
Study population. ED: emergency department. EMR: electronic medical record.

There were 147,865 adult ED encounters from January 1, 2016, to December 31, 2018. After excluding patients with cardiac arrest or death upon ED arrival (n=401), those transferred to another hospital (n=6230), discharged with uncompleted care (n=2696), and with missing or invalid values (n=58,105), a total of 80,433 ED adult patients were included in this study, with 3737 (4.6%) of them identified as experiencing critical care (Figure 1).

The study population of this study was split into two samples: (1) a training data set, comprising 80% of the data set, with 64,346 patients and containing 3015 (4.7%) critical care patients, and (2) a validation data set, consisting of the remaining 20% of the data set, with 16,807 patients, including 722 (4.5%) of them ascertained as receiving critical care. The characteristics of the training and validation data sets were not significantly different (Table S3, Multimedia Appendix 1).

The characteristics of the ED patients according to the study outcome are presented in Table 1. Critically ill patients were more likely to be female, older, call EMS, and have a higher proportion of illness than those without critical care. The time interval between onset and ED arrival was not significantly different between patients with and without critical care. Initial vital signs and levels of consciousness were significantly different between the two groups. The most common chief complaint among critically ill patients was dyspnea and fever among those without critical care. The median of KTAS at ED triage was 2 points (emergent level) for the critical care group and 3 points (urgent level) for the noncritical care group. The ED length of stay of patients was 6.4 hrs in the critical care group and 4.0 hrs in the noncritical care group (Table 1).

**Table 1:** Baseline characteristics of adult emergency patients according to critical care.

Characteristic			Total (N=80,433)		ED^a^ discharge (n=76,696)		Critical care (n=3737)		*P* value	
**Gender, n (%)**										<.001
	Male	39,210 (48.7)		37,010 (48.3)		2200 (58.9)				
	Female	41,223 (51.3)		39,686 (51.7)		1537 (41.1)				
Age, median (IQR)			61.0 (46.0-73.0)		61.0 (45.0-72.0)		69.0 (58.0-77.0)		<.001	
Interval between onset and ED arrival (hour), median (IQR)			23.9 (3.8-96.0)		23.9 (3.8-96.0)		23.1 (4.4-95.8)		.17	
Mode of ED arrival (EMS^b^ use), n (%)			19,264 (24.0)		17,162 (22.4)		2102 (56.2)		<.001	
**Reason for ED visit, n (%)**										<.001
	Illness	73,645 (91.6)		70,021 (91.3)		3624 (97.0)				
	Injury	6788 (8.4)		6675 (8.7)		113 (3.0)				
**Initial vital sign data, median (IQR)**										
	SBP^c^, mmHg	141.0 (126.0-165.0)		142.0 (126.0-165.0)		133.0 (113.0-160.0)		<.001		
	DBP^d^, mmHg	81.0 (72.0-92.0)		82.0 (72.0-92.0)		75.0 (63.0-88.0)		<.001		
	PR^e^, beats/min	86.0 (74.0-101.0)		86.0 (74.0-101.0)		94.0 (77.0-112.0)		<.001		
	RR^f^, breaths/min	18.0 (16.0-20.0)		18.0 (16.0-20.0)		20.0 (18.0-24.0)		<.001		
	BT^g^, °C	36.5 (36.3-36.7)		36.5 (36.3-36.7)		36.5 (36.3-37.0)		<.001		
	SpO_2_^h^, %	97.0 (96.0-98.0)		97.0 (96.0-98.0)		97.0 (94.0-98.0)		<.001		
Nonalert, n (%)			3592 (4.5)		2858 (3.7)		734 (19.6)		<.001	
**Chief complaint, n (%)**										<.001
	Dyspnea	7705 (9.6)		6793 (8.9)		912 (24.4)				
	Fever	7275 (9.0)		6991 (9.1)		284 (7.6)				
	Abdominal pain	5302 (6.6)		5136 (6.7)		166 (4.4)				
	Chest pain	5042 (6.3)		4487 (5.9)		555 (14.9)				
	Dizziness	3550 (4.4)		3505 (4.6)		45 (1.2)				
	Others	51,559 (64.1)		49,784 (64.9)		1775 (47.5)				
**KTAS^i^ level, n (%)**										<.001
	1: Resuscitation	870 (1.1)		404 (0.5)		466 (12.5)				
	2: Emergent	12,646 (15.7)		10,692 (13.9)		1954 (52.3)				
	3: Urgent	47,977 (59.6)		46,702 (60.9)		1275 (34.1)				
	4: Less urgent	16,637 (20.7)		16,599 (21.6)		38 (1.0)				
	5: Nonurgent	2303 (2.9)		2299 (3.0)		4 (0.1)				
ED LOS^j^ (hour), median (IQR)			4.1 (2.4-7.3)		4.0 (2.4-7.2)		6.4 (3.7-10.4)		<.001	
**ED disposition, n (%)**										<.001
	ED discharge	57,014 (70.9)		57,014 (74.3)		0 (0.0)				
	Ward admission	19,123 (23.8)		18,630 (24.3)		493 (13.2)				
	ICU^k^ admission	3170 (3.9)		0 (0.0)		3170 (84.8)				
	OR^l^ admission	1080 (1.3)		1052 (1.4)		28 (0.7)				
	ED mortality	46 (0.1)		0 (0.0)		46 (1.2)				
In-hospital mortality, n (%)			804 (1.0)		0 (0.0)		804 (21.5)		<.001	

^a^ED: emergency department.

^b^EMS: emergency medical service.

^c^SBP: systolic blood pressure.

^d^DBP: diastolic blood pressure.

^e^PR: pulse rate.

^f^RR: respiratory rate.

^g^BT: body temperature.

^h^SpO_2_: oxygen saturation.

^i^KTAS: Korean Triage and Acute Scale.

^j^LOS: length of stay.

^k^ICU: intensive care unit.

^l^OR: operating room.

### Main Analysis

**Figure 2:**
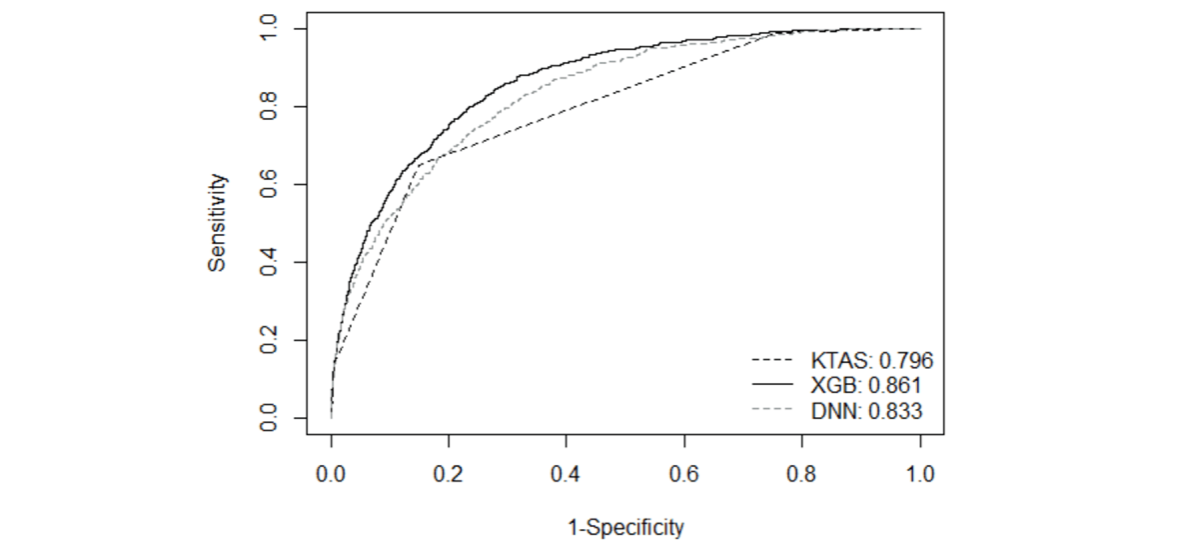
Area under the receiver operating characteristic curve for validation data set. DNN: deep neural network; KTAS: Korean Triage and Acute Scale; XGB: extreme gradient boosting.

**Figure 3:**
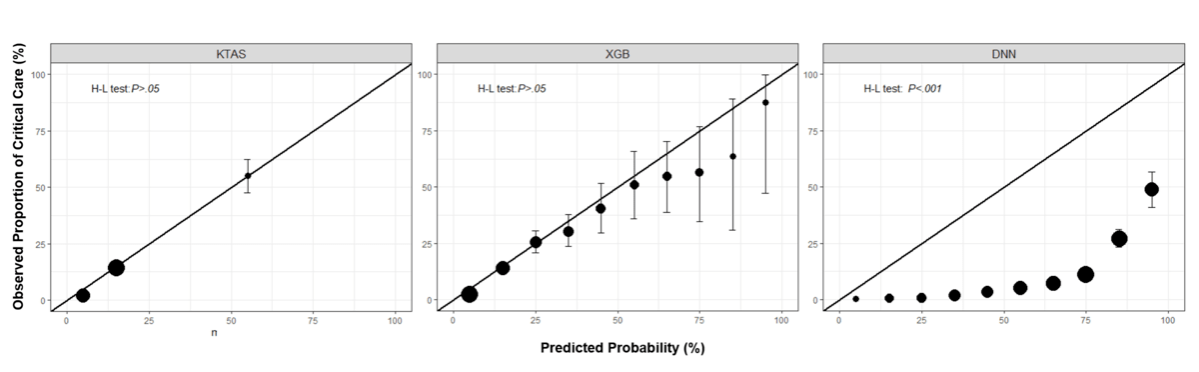
Calibration plot for validation data set. DNN: deep neural network; H-L test: Hosmer-Lemeshow test; KTAS: Korean Triage and Acute Scale; XGB: extreme gradient boosting. The observed probability of critical care with 95% CI is plotted against predicted probability by 10% interval. The diagonal line, which is represented as ideal, means perfect prediction. Point size indicates the relative number of observations in each bin.

Classification results for the validation data set are presented in Table 2. While the baseline model with a single variable of KTAS had the lowest discriminative ability of AUROC 0.796 (95% CI 0.781-0.811), the machine learning models had higher discriminative ability. When using triage information, age, gender, mode of ED arrival, the time interval between onset and ED arrival, reason of ED visit, chief complaints, the six vital sign measurements, and level of consciousness, the XGB algorithm yielded a higher AUROC of 0.861 (95% CI 0.848-0.874) than DNN of 0.833 (95% CI 0.819-0.848) for the validation data set. The machine learning models achieved higher reclassification improvement over the reference model with positive NRI *(P<*.05). As Figure 2 depicted, the AUROCs between the models with the full set of variables and the baseline model were significantly different. (DeLong’s test for the validation data set: *P*<.05) The XGB model showed good calibration (Hosmer–Lemeshow test for the validation data set: *P*>.05), and calibration of the DNN model was poor with *P*<.001. The calibration plots on the validation data set were illustrated in Figure 3. We selected the XGB model as the final model in this study, considering discrimination, net reclassification, and calibration.

The predictive performance metrics of the validation cohort, including sensitivity, specificity, PPV, and NPV, are presented in Table 3. The XGB and DNN model showed a higher sensitivity of 0.85 than the baseline model (0.65, 95% CI 0.61-0.68) with a cutoff at the level of KTAS 2. As a trade-off, the specificity of the conventional model using a single variable of KTAS had a higher specificity of 0.85 (95% CI 0.84-0.86) than that of the XGB model at 0.71 (95% CI 0.70-0.72) and the DNN model at 0.64 (95% CI 0.64-0.65). Due to the low prevalence of critical care outcomes, all models had high NPV with a 95% CI ranging from 0.98 to 0.99.

The number of the actual and predicted outcomes according to the level of KTAS is provided in Table 4. For the validation data set, the baseline model correctly identified 469 patients needing critical care in triage levels 1 and 2, which accounted for 65.0% of all critical care outcomes. However, it overtriaged 2296 patients in these high acuity categories. Undertriaging 35% of patients in need of critical care, the conventional model using a single variable of KTAS failed to predict all critical care outcomes (253 cases) for triage levels 3 to 5. Compared to the baseline model, the XGB model reduced false-positive cases from 2296 to 1533 in KTAS levels 1 and 2 and the false-negative cases from 253 to 80 in KTAS levels 3 to 5.

**Table 2:** Discrimination, reclassification, and calibration of critical care outcome prediction models for the validation cohort.

Model	Discrimination		Reclassification			Calibration
	AUROC^a^ (95% CI)	*P* value^b^	NRI^c^ (95% CI)	*P* value	H-L^d^ test, *P* value	
KTAS^e^	0.796 (0.781-0.811)	Reference	Reference	Reference	.80	
XGB^f^	0.861 (0.848-0.874)	<.001	0.293 (0.219-0.366)	<.001	.24	
DNN^g^	0.833 (0.819-0.848)	<.001	0.032 (0.024-0.041)	<.001	<.001	

^a^AUROC: area under the receiver operating characteristic.

^b^*P* value for AUROC was calculated using DeLong’s test.

^c^NRI: net reclassification index.

^d^H-L: Hosmer-Lemeshow test.

^e^KTAS: Korean Triage and Acute Scale.

^f^XGB: extreme gradient boosting.

^g^DNN: deep neural network.

**Table 3:** Performance of critical care outcome prediction models in validation cohorts.

Model	Cutoff score	TP^a^	FP^b^	TN^c^	FN^d^	Sensitivity (95% CI)	Specificity (95% CI)	PPV^e^ (95% CI)	NPV^f^ (95% CI)
Baseline KTAS^g^	0.156^h^	469	2296	13,069	253	0.65 (0.61-0.68)	0.85 (0.84-0.86)	0.17 (0.16-0.18)	0.98 (0.98-0.98)
XGB^i^	0.036	616	4476	10,889	106	0.85 (0.83-0.88)	0.71 (0.70-0.72)	0.12 (0.11-0.13)	0.99 (0.99-0.99)
DNN^j^	0.444	614	5475	9890	108	0.85 (0.82-0.88)	0.64 (0.64-0.65)	0.10 (0.09-0.11)	0.99 (0.99-0.99)

^a^TP: true positive.

^b^FP: false positive.

^c^TN: true negative.

^d^FN: false negative.

^e^PPV: positive predictive values.

^f^NPV: negative predictive values.

^g^KTAS: Korean Triage and Acute Scale.

^h^Cutoff probability of 0.156 for the baseline model by logistic regression corresponds to KTAS score of 2.

^i^XGB: extreme gradient boosting.

^j^DNN: deep neural network.

**Table 4:** The performance comparison of prediction models in validation cohorts according to the level of KTAS.

KTAS^a^ level	Actual critical care, n (%)	Baseline model					XGB^b^ model			
		TP^c^	FP^d^	TN^e^	FN^f^	TP		FP	TN	FN
1: Resuscitation (n=178, 1.1%)	98 (13.6)	98	80	0	0	96		77	3	2
2: Emergent (n=2587, 16.1%)	371 (51.4)	371	2216	0	0	347		1456	760	24
3: Urgent (n=9559, 59.4%)	244 (33.8)	0	0	9315	244	170		2622	6693	74
4: Less urgent (n=3312, 20.6%)	9 (1.2)	0	0	3303	9	3		297	3006	6
5: Nonurgent (n=451, 2.8%)	0 (0.0)	0	0	451	0	0		24	427	0
Total (n=16,086, 100%)	722 (100)	469	2296	13,069	253	616		4476	10,889	106

^a^KTAS: Korean Triage and Acute Scale.

^b^XGB: extreme gradient boosting.

^c^TP: true positive.

^d^FP: false positive.

^e^TN: true negative.

^f^FN: false negative.

### Variable Importance and Partial Dependence Plot

**Figure 4:**
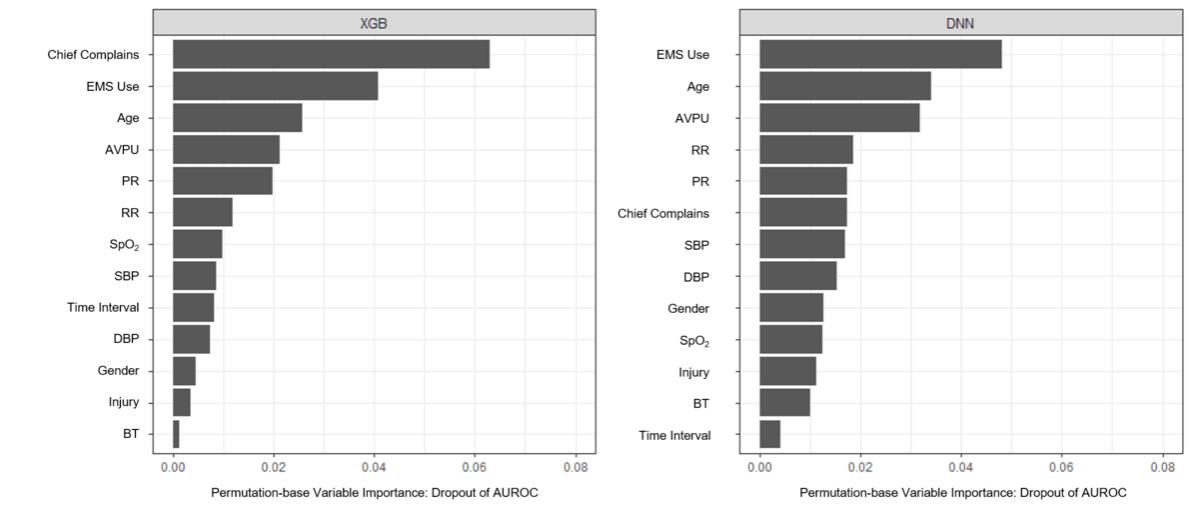
Feature importance. The time interval denotes the time between onset and ED arrival. AUROC: area under the receiver operating characteristic curve; AVPU: alert, verbal, painful, and unresponsive; BT: body temperature; DBP: diastolic blood pressure; DNN: deep neural network; ED: emergency department; EMS: emergency medical service; PR: pulse rate; RR: respiratory rate; SBP: systolic blood pressure; SpO_2_: oxygen saturation; XGB: extreme gradient boosting.

We computed permutation-based variable importance for the XGB and DNN model in Figure 4. The variable ranked as a top priority was chief complaints for the XGB model and EMS use for the DNN model. Despite the ranking difference in variable importance between the XGB and DNN models, variables higher in the list, including chief complaints, EMS use, age, AVPU, PR, and RR, were identical.

**Figure 5:**
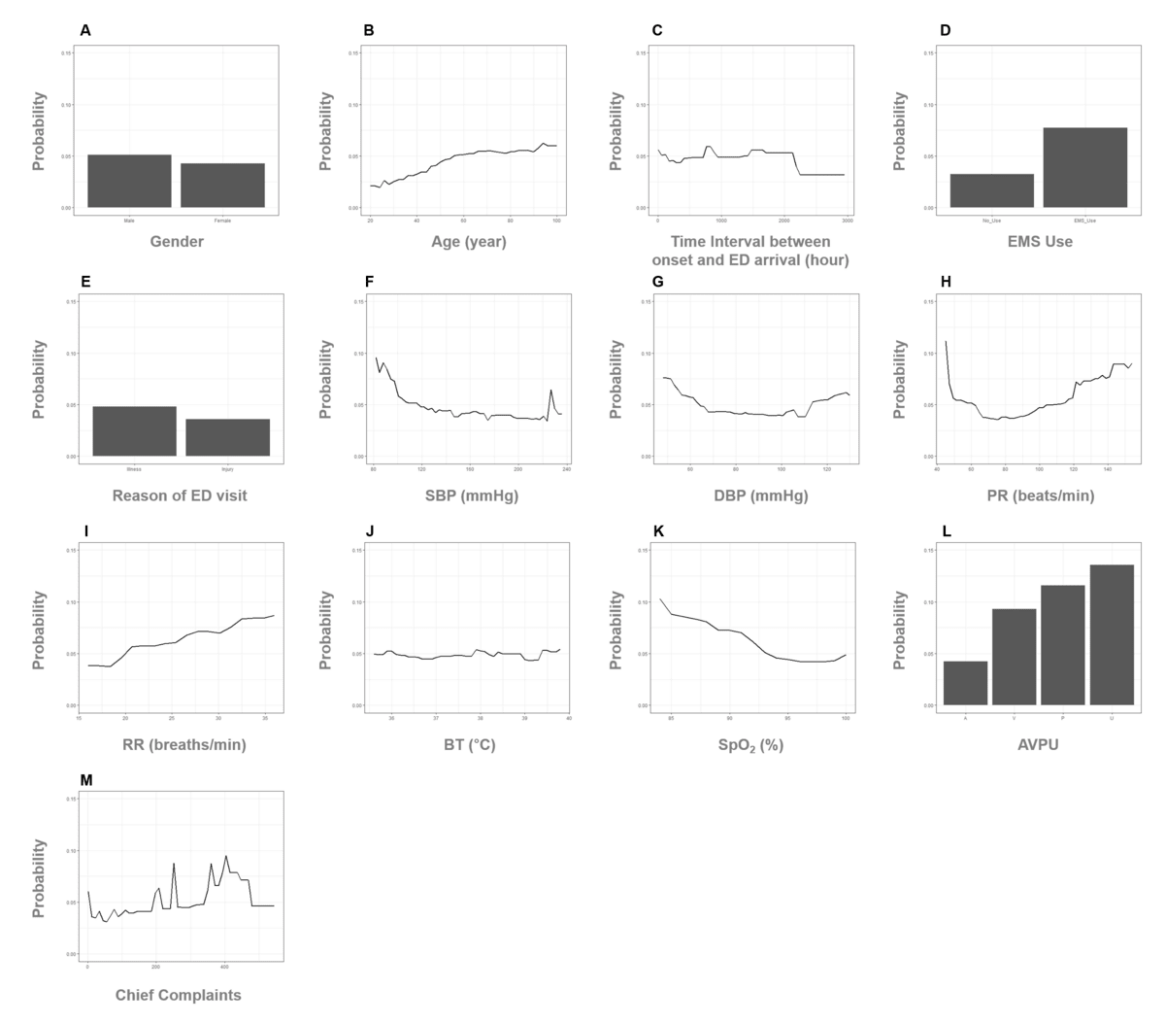
Partial dependence plot. A. gender, B. age, C. time interval between onset and ED arrival, D. EMS use, E. reason of ED visit, F. SBP, G. DBP, H. PR, I. RR, J. BT, K. SpO_2_, L. AVPU, and M. chief complaints. The partial dependence plot shows the marginal effect of a single variable on the prediction outcome; the value of the y-axis is the predicted probability for critical care. AVPU: alert, verbal, pain, and unresponsive; BT: body temperature; DBP: diastolic blood pressure; ED: emergency department; PR: pulse rate; RR: respiratory rate; SBP: systolic blood pressure; SpO_2_: oxygen saturation; XGB: extreme gradient boosting.

For the XGB model defined as the final prediction model, the relationship between each variable and the prediction outcome for the validation data set is illustrated in Figure 5. The PDP shows the marginal effect of a single variable on the prediction outcome. The value of the y-axis on PDP is the predicted probability for critical care. The nonlinear associations of all vital sign variables to critical outcome predictions were demonstrated. For age, RR, and SpO_2_, we found the pattern of the critical care prediction in the XGB model, indicating the probability of being classified as patients in need of critical care increased with older age, higher RR, and lower SpO_2_. For SBP, DBP, and PR, we observed a U-shaped relationship between each vital sign and the critical care prediction.

## Discussion

### Principal Findings

In this study, based on the data of 80,433 ED adult patients, we applied two modern machine learning approaches (ie, XGB and DNN) to the routinely collected triage information (age, gender, mode of ED arrival, the time interval between onset and ED arrival, reason of ED visit, chief complaints, six vital signs, and level of consciousness) for the critical care outcome prediction in ED. The prediction models demonstrated superior performance of discrimination from AUROC 0.833 to AUROC 0.861 for the validation cohort and net reclassification compared to the conventional baseline model using KTAS (AUROC 0.796). The XGB model showed better discriminating power (AUROC 0.861) than the DNN model. We revealed that the XGB model was well-calibrated in predicting critical care outcomes (Hosmer-Lemeshow test; *P*>.05).

The objective of this study was to accurately differentiate high-risk patients from the less urgent patients at the triage stage in the ED. Expedited evaluation and ED care of patients with critical illnesses are crucial for maximizing clinical outcomes, providing a strong rationale for their prediction at triage [7,42]. Previous studies have documented that current five-level triage systems (eg, ESI, MTS, and KTAS) have a suboptimal ability to identify patients at high risk, low inter-rater agreement, and high variability within the same triage level [4,6-10]. Hence, machine learning models incorporating variables of demographics, mode of ED arrival, chief complaints, and vital signs extracted from triage information have been investigated to support accurate and rapid decision-making of ED clinicians. This study extends the earlier research. The discriminative performance gains of the critical care outcome prediction were obtained from the XGB algorithm, which has the excellence to handle nonlinear interactions between variables and the prediction outcome.

In this study, a large number (85.5%) of the patients without a need of critical care were classified into KTAS levels 3 to 5 (83.2% of the entire population), while the majority (64.8%) of the critically ill patient group was assigned into KTAS level 1 and 2 (16.8 % of all patients). We demonstrated that the XGB model correctly detected critically ill patients who were undertriaged into lower-acuity KTAS levels 3 to 5 in the baseline model. The ability to reduce false-negative cases provides a strong rationale for adopting the machine learning algorithm model at ED triage, where the accurate and rapid identification of patients at high risk is a matter of the utmost importance. Furthermore, we observed that the XGB model reduced the number of false-positive cases that were overtriaged into high-acuity levels 1 to 2 in the baseline model, which may prevent excessive resource utilization in ED practices.

This research proved that the XGB model had agreement between the predicted probability and the observed proportion of critical care occurrences. The calibration plot in Figure 3 visualized how well the forecast probabilities from the XGB model were calibrated. Despite the importance of calibration in the prediction model to support clinician decision, systematic reviews have found that calibration is assessed far less than discrimination [24,25,27], which is problematic since poor calibration can make predictions misleading [24,26-28]. Machine learning algorithms are vulnerable to overfitting [24,33,43]. Due to overfitting, most machine learning algorithms, especially neural networks, are known to produce poor calibration when validated with new data [24,33,44,45]. However, XGB controls the model complexity by embedding a regularization term into the objective function to avoid overfitting [40,46,47]. Our findings suggest that the probabilities of the XGB model for predicting patients at high risk in ED were reliable.

Explaining the predictions of block-box machine learning has become highlighted. For the global interpretation of the model, we visualized the nonlinear relationship between a variable and outcome results in predicting critically ill patients using PDPs (Figure 5). The XGB algorithm interpreted that, on average, higher RR and lower SpO_2_ are associated with a high probability of critical care outcomes, and there was a U-shaped relationship between SBP, DBP, and PR and the outcome results. The interpretation of the XGB model clearly reflected the characteristics of vital signs and was in line with medical knowledge. There are several interpretation techniques for global and local levels of machine learning interpretation. A future study of the multilevel interpretation of machine learning algorithm predictions is warranted.

Using triage information and the XGB algorithm, the artificial intelligent model for predicting patients at high risk in this study can be implemented in the ED setting without additional burden, which may support prompt and accurate clinician decision-making at the early stage of ED triage, leading to the improvement of patients’ health outcomes and contributing to efficient ED resource allocation.

### Limitations

This study has several limitations. First, we used the data from a single ED of a tertiary-care university hospital; therefore, external validation is needed for the generalization of the results. Second, this study did not address how the prediction model could be deployed into the clinical pathway; therefore, future studies applying the prediction model during triage are warranted.

### Conclusions

This study demonstrated that using initial triage information routinely collected in the ED, the machine learning model improved the discrimination and net reclassification for predicting patients in need of critical care in ED compared to the conventional approach with KTAS. Moreover, we demonstrated that the XGB model was well-calibrated and interpreted nonlinear characteristics of vital sign predictors in line with medical knowledge.

## Appendix

Multimedia Appendix 1 Supplementary files including (1) variable description for clinical outcome prediction in emergency department, (2) hyperparameter optimization, and (3) comparison of baseline characteristics of study population between training and validation datasets.

## Abbreviations

AUROC: area under the receiver operating characteristic
AVPU: alert, verbal, painful, and unresponsive
BT: body temperature
CTAS: Canadian Triage and Acuity Scale
DBP: diastolic blood pressure
DNN: deep neural network
ED: emergency department
ESI: emergency severity index
ICU: intensive care unit
KTAS: Korean Triage and Acute Scale
MTS: Manchester Triage System
NPV: negative predictive value
NRI: net reclassification index
PDP: partial dependence plot
PPV: positive predictive value
PR: pulse rate
RR: respiratory rate
SBP: systolic blood pressure
XGB: extreme gradient boosting

## References

[1] Morley C, Unwin M, Peterson GM, Stankovich J, Kinsman L (2018). Emergency department crowding: A systematic review of causes, consequences and solutions. PLoS One.

[2] Zachariasse JM, van der Hagen V, Seiger N, Mackway-Jones K, van Veen M, Moll HA (2019). Performance of triage systems in emergency care: a systematic review and meta-analysis. BMJ Open.

[3] Graham B, Bond R, Quinn M, Mulvenna M (2018). Using Data Mining to Predict Hospital Admissions From the Emergency Department. IEEE Access.

[4] Dugas AF, Kirsch TD, Toerper M, Korley F, Yenokyan G, France D, Hager D, Levin S (2016). An Electronic Emergency Triage System to Improve Patient Distribution by Critical Outcomes. J Emerg Med.

[5] Fernandes M, Vieira SM, Leite F, Palos C, Finkelstein S, Sousa JM (2020). Clinical Decision Support Systems for Triage in the Emergency Department using Intelligent Systems: a Review. Artif Intell Med.

[6] Fernandes M, Mendes R, Vieira SM, Leite F, Palos C, Johnson A, Finkelstein S, Horng S, Celi LA (2020). Predicting Intensive Care Unit admission among patients presenting to the emergency department using machine learning and natural language processing. PLoS One.

[7] Levin S, Toerper M, Hamrock E, Hinson JS, Barnes S, Gardner H, Dugas A, Linton B, Kirsch T, Kelen G (2018). Machine-Learning-Based Electronic Triage More Accurately Differentiates Patients With Respect to Clinical Outcomes Compared With the Emergency Severity Index. Ann Emerg Med.

[8] Fernandes M, Mendes R, Vieira SM, Leite F, Palos C, Johnson A, Finkelstein S, Horng S, Celi LA (2020). Risk of mortality and cardiopulmonary arrest in critical patients presenting to the emergency department using machine learning and natural language processing. PLoS One.

[9] Raita Y, Goto T, Faridi MK, Brown DFM, Camargo CA, Hasegawa K (2019). Emergency department triage prediction of clinical outcomes using machine learning models. Crit Care.

[10] Goto T, Camargo CA, Faridi MK, Yun BJ, Hasegawa K (2018). Machine learning approaches for predicting disposition of asthma and COPD exacerbations in the ED. Am J Emerg Med.

[11] Choi SW, Ko T, Hong KJ, Kim KH (2019). Machine Learning-Based Prediction of Korean Triage and Acuity Scale Level in Emergency Department Patients. Healthc Inform Res.

[12] Kwon J, Lee Y, Lee Y, Lee S, Park H, Park J (2018). Validation of deep-learning-based triage and acuity score using a large national dataset. PLoS One.

[13] Lee JH, Park YS, Park IC, Lee HS, Kim JH, Park JM, Chung SP, Kim MJ (2019). Over-triage occurs when considering the patient's pain in Korean Triage and Acuity Scale (KTAS). PLoS One.

[14] Lundberg SM, Nair B, Vavilala MS, Horibe M, Eisses MJ, Adams T, Liston DE, Low DK, Newman S, Kim J, Lee S (2018). Explainable machine-learning predictions for the prevention of hypoxaemia during surgery. Nat Biomed Eng.

[15] Elshawi R, Al-Mallah MH, Sakr S (2019). On the interpretability of machine learning-based model for predicting hypertension. BMC Med Inform Decis Mak.

[16] Mahmoudi E, Kamdar N, Kim N, Gonzales G, Singh K, Waljee AK (2020). Use of electronic medical records in development and validation of risk prediction models of hospital readmission: systematic review. BMJ.

[17] Rahimian F, Salimi-Khorshidi G, Payberah AH, Tran J, Ayala Solares R, Raimondi F, Nazarzadeh M, Canoy D, Rahimi K (2018). Predicting the risk of emergency admission with machine learning: Development and validation using linked electronic health records. PLoS Med.

[18] Goto T, Camargo CA, Faridi MK, Freishtat RJ, Hasegawa K (2019). Machine Learning-Based Prediction of Clinical Outcomes for Children During Emergency Department Triage. JAMA Netw Open.

[19] Hong WS, Haimovich AD, Taylor RA (2018). Predicting hospital admission at emergency department triage using machine learning. PLoS One.

[20] Zlotnik A, Alfaro MC, Pérez María Carmen Pérez, Gallardo-Antolín Ascensión, Martínez Juan Manuel Montero (2016). Building a Decision Support System for Inpatient Admission Prediction With the Manchester Triage System and Administrative Check-in Variables. Comput Inform Nurs.

[21] Alba AC, Agoritsas T, Walsh M, Hanna S, Iorio A, Devereaux PJ, McGinn T, Guyatt G (2017). Discrimination and Calibration of Clinical Prediction Models: Users' Guides to the Medical Literature. JAMA.

[22] Steyerberg EW, Vergouwe Y (2014). Towards better clinical prediction models: seven steps for development and an ABCD for validation. Eur Heart J.

[23] Steyerberg E, Vickers AJ, Cook NR, Gerds T, Gonen M, Obuchowski N, Pencina MJ, Kattan MW (2010). Assessing the performance of prediction models: a framework for traditional and novel measures. Epidemiology.

[24] Van Calster B, McLernon DJ, van Smeden M, Wynants L, Steyerberg EW, Topic Group ‘Evaluating diagnostic testsprediction models’ of the STRATOS initiative (2019). Calibration: the Achilles heel of predictive analytics. BMC Med.

[25] Christodoulou E, Ma J, Collins GS, Steyerberg EW, Verbakel JY, Van Calster B (2019). A systematic review shows no performance benefit of machine learning over logistic regression for clinical prediction models. J Clin Epidemiol.

[26] Wessler BS, Paulus J, Lundquist CM, Ajlan M, Natto Z, Janes WA, Jethmalani N, Raman G, Lutz JS, Kent DM (2017). Tufts PACE Clinical Predictive Model Registry: update 1990 through 2015. Diagn Progn Res.

[27] Collins GS, de Groot JA, Dutton S, Omar O, Shanyinde M, Tajar A, Voysey M, Wharton R, Yu L, Moons KG, Altman DG (2014). External validation of multivariable prediction models: a systematic review of methodological conduct and reporting. BMC Med Res Methodol.

[28] Van Calster B, Vickers AJ (2015). Calibration of risk prediction models: impact on decision-analytic performance. Med Decis Making.

[29] Tonekaboni S (2019). What Clinicians Want: Contextualizing Explainable Machine Learning for Clinical End Use. Proceedings of the 4th Machine Learning for Healthcare Conference, PMLR 106:359-380, 2019.

[30] Ogunleye A, Wang Q (2020). XGBoost Model for Chronic Kidney Disease Diagnosis. IEEE/ACM Trans Comput Biol and Bioinf.

[31] Huang Z, Hu C, Chi C, Jiang Z, Tong Y, Zhao C (2020). An Artificial Intelligence Model for Predicting 1-Year Survival of Bone Metastases in Non-Small-Cell Lung Cancer Patients Based on XGBoost Algorithm. Biomed Res Int.

[32] Spangler D, Hermansson T, Smekal D, Blomberg H (2019). A validation of machine learning-based risk scores in the prehospital setting. PLoS One.

[33] Hou C, Zhong X, He P, Xu B, Diao S, Yi F, Zheng H, Li J (2020). Predicting Breast Cancer in Chinese Women Using Machine Learning Techniques: Algorithm Development. JMIR Med Inform.

[34] Huang Y, Li W, Macheret F, Gabriel RA, Ohno-Machado L (2020). A tutorial on calibration measurements and calibration models for clinical prediction models. J Am Med Inform Assoc.

[35] Cava WL, Bauer C, Moore JH, Pendergrass SA (2019). Interpretation of machine learning predictions for patient outcomes in electronic health records. AMIA Annu Symp Proc.

[36] Zhou W, Wang Y, Gu X, Feng Z, Lee K, Peng Y, Barszczyk A (2021). Importance of general adiposity, visceral adiposity and vital signs in predicting blood biomarkers using machine learning. Int J Clin Pract.

[37] Muhlestein W, Akagi D, Kallos J, Morone P, Weaver K, Thompson R, Chambless L (2018). Using a Guided Machine Learning Ensemble Model to Predict Discharge Disposition following Meningioma Resection. J Neurol Surg B Skull Base.

[38] Gómez-Ramírez J, Ávila-Villanueva M, Fernández-Blázquez MA (2020). Selecting the most important self-assessed features for predicting conversion to mild cognitive impairment with random forest and permutation-based methods. Sci Rep.

[39] Delfin C, Krona H, Andiné P, Ryding E, Wallinius M, Hofvander B (2019). Prediction of recidivism in a long-term follow-up of forensic psychiatric patients: Incremental effects of neuroimaging data. PLoS One.

[40] Rzychoń M, Żogała A, Róg L (2021). Experimental study and extreme gradient boosting (XGBoost) based prediction of caking ability of coal blends. Journal of Analytical and Applied Pyrolysis.

[41] Roger E, Torlay L, Gardette J, Mosca C, Banjac S, Minotti L, Kahane P, Baciu M (2020). A machine learning approach to explore cognitive signatures in patients with temporo-mesial epilepsy. Neuropsychologia.

[42] Elliott DJ, Williams KD, Wu P, Kher HV, Michalec B, Reinbold N, Coletti CM, Patel BJ, Dressler RM (2015). An Interdepartmental Care Model to Expedite Admission from the Emergency Department to the Medical ICU. Jt Comm J Qual Patient Saf.

[43] Zhang Z, Ho KM, Hong Y (2019). Machine learning for the prediction of volume responsiveness in patients with oliguric acute kidney injury in critical care. Crit Care.

[44] Kull Meelis, Perello-Nieto Miquel, Kängsepp Markus, Silva Filho Telmo, Song Hao, Flach Peter (2019). Beyond temperature scaling: Obtaining well-calibrated multi-class probabilities with Dirichlet calibration. Advances in Neural Information Processing Systems 32 (NeurIPS 2019).

[45] Guo C (2017). On calibration of modern neural networks. Proceedings of the 34th International Conference on Machine Learning in Proceedings of Machine Learning Research 70: PMLR, 2017.

[46] Do DT, Le NQK (2020). Using extreme gradient boosting to identify origin of replication in Saccharomyces cerevisiae via hybrid features. Genomics.

[47] Wu T, Chen H, Jhou M, Chen Y, Chang T, Lu C (2020). Evaluating the Effect of Topical Atropine Use for Myopia Control on Intraocular Pressure by Using Machine Learning. J Clin Med.

