# Supplementary material for: Prediction of Critical Care Outcome for Adult Patients Presenting to Emergency Department Using Initial Triage Information: An XGBoost Algorithm Analysis
Source: JMIR Med Inform. 2021 Sep 20;9(9):e30770. doi: 10.2196/30770 (PMC8491120; doi:10.2196/30770)
Supplement: Multimedia Appendix 1 [file medinform_v9i9e30770_app1.docx]

Supplementary Table 1. Variable description for clinical outcome prediction in emergency department

| Name | Description | Type | Preprocessing |
| --- | --- | --- | --- |
| Gender | Sex of Patient | Categorical | Male (0), Female (1) |
| Age | Age in terms of years | Continuous | - |
| Onset_Interval | Time interval between onset and ED arrival by hour | Continuous | - |
| EMS_USE | Mode of ED arrival | Categorical | ED arrival without ems use (0), with ems use (1) |
| Injury | Reason of ED visiting | Categorical | Illness (0), Injury (1) |
| SBP | Systolic blood pressure (mm Hg) at ED triage | Continuous | Excluded as invalid value if not in 20 < SBP < 300* |
| DBP | Diastolic blood pressure (mm Hg) at ED triage | Continuous | Excluded as invalid value if not in 5 < DBP < 200* |
| PR | Pulse rate at ED triage (beats/min) | Continuous | Excluded as invalid value if not in 1 < PR ≤ 300* |
| RR | Respiratory rate (breaths/min) at ED triage | Continuous | Excluded as invalid value if not in 1 ≤ RR ≤ 80* |
| BT | Body temperature (°C) at ED triage | Continuous | Excluded as invalid value if not in 20 ≤ BT ≤ 45* |
| SpO_2_ | Oxygen Saturation at ED triage | Continuous | Excluded as invalid value if not in 50 < SpO_2_ ≤ 100* |
| AVPU | Level of consciousness | Categorical | Alert (0), Verbal (1), Painful (2), Unresponsive (3) |
| Chief complaint | There were 547 chief complaint codes during the study period. The codes was sorted by frequency and was transformed as number. | Categorical | Number of chief complaint code from 1 to547  : 1 (most frequent code) ⭤ (least frequent) 547 |
| KTAS | Korean Triage and Acute Scale | Categorical | Immediate (1), Very urgent (2), Urgent (3),  Less urgent (4), Non-urgent (5) |

(Reference) *Fernandes M, Mendes R, Vieira SM, Leite F, Palos C, Johnson A, et al. Risk of mortality and cardiopulmonary arrest in critical patients presenting to the emergency department using machine learning and natural language processing. PloS one. 2020;15(4):e0230876-e.

Raita Y, Goto T, Faridi MK, Brown DFM, Camargo CA, Jr., Hasegawa K. Emergency department triage prediction of clinical outcomes using machine learning models. Critical care (London, England). 2019;23(1):64-.

Supplementary Table 2. Hyperparameter optimization

| Algorithm | Hyperparameter |
| --- | --- |
| LR | No hyperparameter tuning is required |
| XGB | Extreme gradient boosting was fitted by R package ‘xgboost’. The grid search for tuning hyperparameters was following:  The maximum depth in the values {1, 2, 3, 4, 5, 6}  The learning rate in the values {0.01, 0.05, 0.1, 0.2, 0.3} |
| DNN | Deep neural network was fitted by R package ‘keras’ with tensorflow backend. The grid search for tuning hyperparameter was following:  The hidden layer investigated in this study was in the values {1, 2, 3, 4, 5}  The lambda for L2 regularization was in the values {0, 0.001, 0.01, 0.1} |

LR: logistic regression; XGB: eXtreme Gradient Boosting; DNN: Deep neural network

Supplementary Table 3. Comparison of baseline characteristics of study population between training and validation datasets

|  | **No. (%) or Median (IQR)** | | | |
| --- | --- | --- | --- | --- |
|  | **Total**  **(N =80,433)** | **Training Dataset**  **(N =64346)** | **Validation Dataset**  **(N =16,087)** | **P-value** |
| Gender |  |  |  | 0.304 |
| Male | 39,210 (48.7%) | 31,309 (48.7%) | 7,901 (49.1%) |  |
| Female | 41,223 (51.3%) | 33,037 (51.3%) | 8,186 (50.9%) |  |
| Age | 61.0 [46.0;73.0] | 61.0 [46.0;73.0] | 61.0 [46.0;73.0] | 0.794 |
| Interval Between Onset and ED arrival (Hour) | 23.9 [ 3.8;96.0] | 23.9 [ 3.8;96.0] | 23.9 [ 3.8;96.0] | 0.997 |
| Mode of ED arrival |  |  |  |  |
| EMS Use | 19,264 (24.0%) | 15,369 (23.9%) | 3,895 (24.2%) | 0.390 |
| Reason for ED visit |  |  |  | 0.946 |
| Illness | 73,645 (91.6%) | 58,913 (91.6%) | 14,732 (91.6%) |  |
| Injury | 6,788 ( 8.4%) | 5,433 ( 8.4%) | 1,355 ( 8.4%) |  |
| Initial vital sign data |  |  |  |  |
| SBP, mmHg | 141.0 [126.0;165.0] | 141.0 [126.0;165.0] | 141.0 [126.0;165.0] | 0.231 |
| DBP, mmHg | 81.0 [72.0;92.0] | 81.0 [72.0;92.0] | 81.0 [71.0;92.0] | 0.329 |
| PR, beats/min | 86.0 [74.0;101.0] | 86.0 [74.0;101.0] | 86.0 [74.0;101.0] | 0.375 |
| RR, breaths/min | 18.0 [16.0;20.0] | 18.0 [16.0;20.0] | 18.0 [16.0;20.0] | 0.207 |
| BT, °C | 36.5 [36.3;36.7] | 36.5 [36.3;36.7] | 36.5 [36.2;36.7] | 0.625 |
| SpO_2_, % | 97.0 [96.0;98.0] | 97.0 [96.0;98.0] | 97.0 [96.0;98.0] | 0.605 |
| Non-Alert | 3592 ( 4.5%) | 2851 ( 4.4%) | 741 ( 4.6%) | 0.346 |
| Chief Complaint |  |  |  | 0.367 |
| Dyspnea | 7,705 ( 9.6%) | 6,188 ( 9.6%) | 1,517 ( 9.4%) |  |
| Fever | 7,275 ( 9.0%) | 5,771 ( 9.0%) | 1,504 ( 9.3%) |  |
| Abdominal Pain | 5,302 ( 6.6%) | 4,273 ( 6.6%) | 1,029 ( 6.4%) |  |
| Chest Pain | 5,042 ( 6.3%) | 4,043 ( 6.3%) | 999 ( 6.2%) |  |
| Dizziness | 3,550 ( 4.4%) | 2,868 ( 4.5%) | 682 ( 4.2%) |  |
| Others | 51,559 (64.1%) | 41,203 (64.0%) | 10,356 (64.4%) |  |
| KTAS level |  |  |  | 0.685 |
| 1: Resuscitation | 870 ( 1.1%) | 692 ( 1.1%) | 178 ( 1.1%) |  |
| 2: Emergent | 12,646 (15.7%) | 10,059 (15.6%) | 2,587 (16.1%) |  |
| 3: Urgent | 47,977 (59.6%) | 38,418 (59.7%) | 9,559 (59.4%) |  |
| 4: Less urgent | 16,637 (20.7%) | 13,325 (20.7%) | 3,312 (20.6%) |  |
| 5: Non-urgent | 2,303 ( 2.9%) | 1,852 ( 2.9%) | 451 ( 2.8%) |  |
| ED LOS (Hour) | 4.1 [ 2.4; 7.3] | 4.1 [ 2.4; 7.3] | 4.2 [ 2.5; 7.4] | 0.095 |
| ED Disposition |  |  |  | 0.380 |
| ED Discharge | 57,014 (70.9%) | 45,680 (71.0%) | 11,334 (70.5%) |  |
| Ward Admission | 19,123 (23.8%) | 15,210 (23.6%) | 3,913 (24.3%) |  |
| ICU Admission | 3,170 ( 3.9%) | 2,551 ( 4.0%) | 619 ( 3.8%) |  |
| OP Room Admission | 1,080 ( 1.3%) | 866 ( 1.3%) | 214 ( 1.3%) |  |
| ED Mortality | 46 ( 0.1%) | 39 ( 0.1%) | 7 ( 0.0%) |  |
| In-hospital Mortality | 804 ( 1.0%) | 656 ( 1.0%) | 148 ( 0.9%) | 0.276 |
| Critical Care | 3,737 ( 4.6%) | 3,015 ( 4.7%) | 722 ( 4.5%) | 0.297 |
| IQR, interquartile range; EMS, emergency medical service; SBP, systolic blood pressure; DBP, diastolic blood pressure; PR, pulse rate; RR, respiratory rate; BT, body temperature; SpO_2_, Oxygen Saturation; KTAS, Korean Triage and Acute Scale; ED, emergency department, LOS, length of stay, ICU, intensive care unit; OP, Operating. | | | | |
